# Anisotropic morphology, formation mechanisms, and fluorescence properties of zirconia nanocrystals

**DOI:** 10.1038/s41598-020-70570-5

**Published:** 2020-08-17

**Authors:** Weiwei Qin, Luyi Zhu

**Affiliations:** 1 Shandong Vocational and Technical University of Engineering, Jinan, 250200 People’s Republic of China; 2 State Key Laboratory of Crystal Materials and Institute of Crystal Materials, Shandong University, Jinan, 250100 People’s Republic of China

**Keywords:** Environmental sciences, Solid Earth sciences

## Abstract

ZrO_2_ nanocrystals with spheres and elongated platelets were systemically prepared through a simple hydrothermal method by the use of ZrOCl_2_·8H_2_O and CH_3_COOK as raw materials. The anisotropic morphology and formation mechanism of the monoclinic and/or tetragonal ZrO_2_ were investigated by X-ray diffraction, Fourier transform infrared spectroscopy, Raman spectroscopy, scanning electron microscope, and high-resolution transmission electron microscope techniques. The uniform elongated platelets and star-like structures were composed of short nanorods with a diameter of approximately 5 nm and a length of approximately 10 nm. The different morphologies were formed due to the different contents of CH_3_COO^−^ and Cl^−^ and their synergy. The fluorescence band position and the band shape remained about the same for excitation wavelengths below 290 nm and the different morphologies of the nanocrystals.

## Introduction

As an important structural and functional material, zirconia (ZrO_2_) has a myriad of technological and commercial applications, such as a catalyst^1^, catalyst support^2,3^, adsorbents^4^, solid oxide fuel^5,6^, and oxygen sensors^7^. Until now, hydro/solvothermal^8^, sol–gel^9^, precipitation^10^, and pulsed plasma in liquid solution^11^ methods have been used to prepare zirconia nanopowders. It has been well known that the physico-chemical properties of the materials not only depended on their chemical compositions and phase structures, but also were related to their morphology, size, exposed facets, surface state, and the like^12–14^. In recent years, the ZrO_2_ nanomaterials with different morphology and sizes were reported, including ZrO_2_ nanoparticles^15,16^, nanofibers^17^, nanobelts^18^, nanowires^19^, nanotubes^20^, flake-like structures^21^, flower- and star-like structures^22,23^, and hollow spheres^24^.

The desired morphologies through the precursor with special structures were obtained during the synthesis of ZrO_2_ nanostructures. For example, a flower-like ZrO_2_ was successfully obtained by hydrothermal treatment of a precursor having a three-dimensional structure at 175 °C for 24 h^22^. Using silanol group-rich mesoporous silica as a hard template, the researchers obtained ordered mesoporous zirconia after soaking, calcining, and removing templates^25^. A zirconium-organic framework was synthesized first, and then mesoporous and tetragonal zirconia were obtained by calcinations^26^. Nano-zirconia with different crystal forms was prepared by microwave heating by changing the ligand type of zirconium-containing precursors^27^. Among the zirconium-containing precursors, acetate and chloride played important roles in the formation of different nanostructures.

In this paper, ZrO_2_ nanoparticles with spheres and elongated platelets were systemically synthesized through a simple hydrothermal method by the use of ZrOCl_2_·8H_2_O and CH_3_COOK as raw materials. The morphology and crystallinity were controlled by reaction conditions. The effects of different molar ratios of the raw materials on the morphologies and growth mechanisms of the nanostructures were investigated. As expected, the different nanostructures of the products lead to novel photoluminescence properties.

## Methods

### Preparation

**Figure 1:**
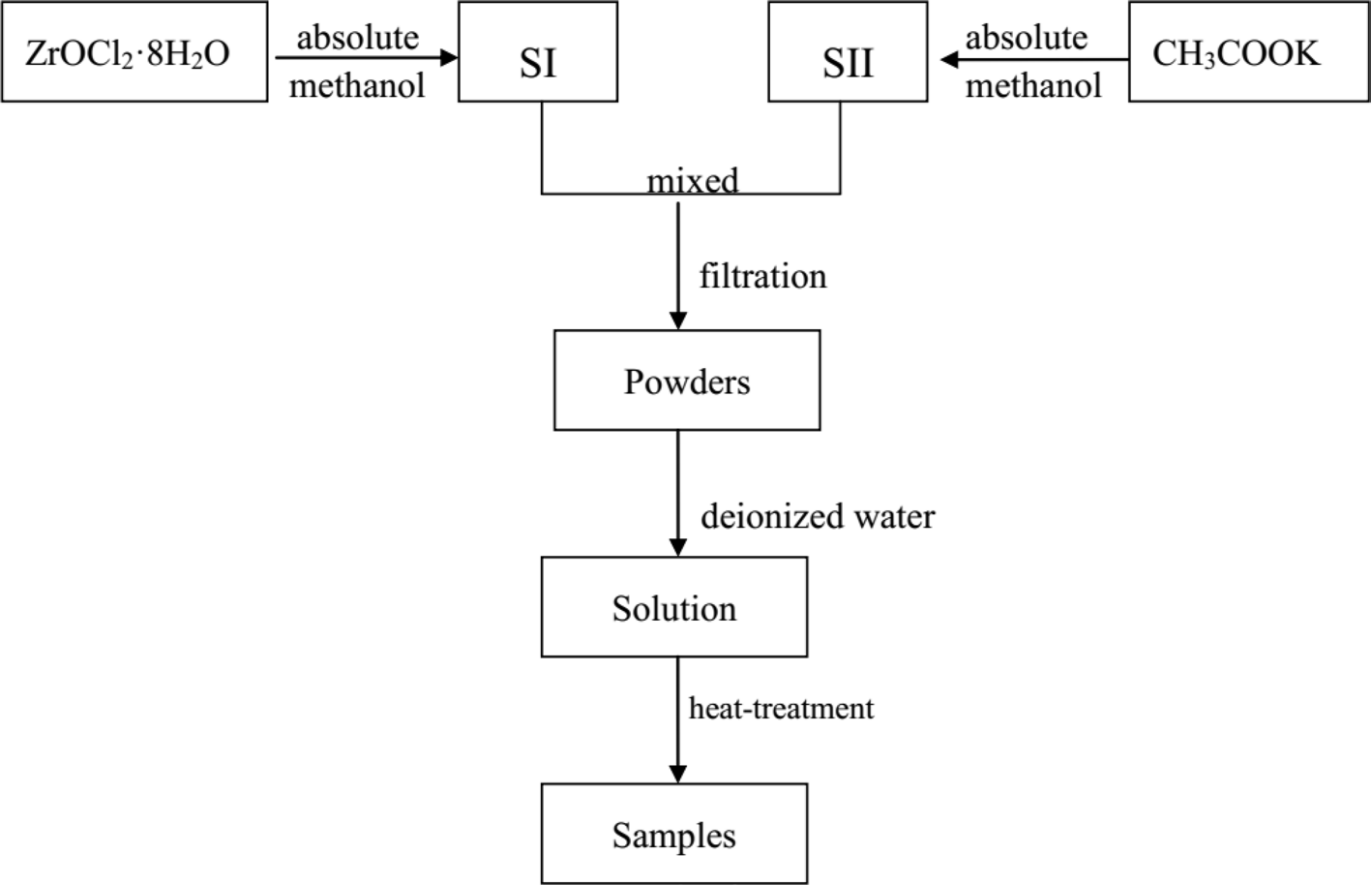
Experimental process for the hydrothermal synthesis of ZrO_2_ nanocrystals.

All reagents were analytical grade and used without further purification. In a typical preparation procedure, as seen the Fig. 1, 3.22 g (0.01 mol) ZrOCl_2_·8H_2_O/(5 ml absolute methanol) and 1.51 g (0.015 mol) CH_3_COOK/(10 ml absolute methanol) were denoted SI and SII, respectively. SII was dropwise added into SI and then the zirconium-containing solution was obtained by filtration, and finally the solution was concentrated into powders. The aforementioned 0.5-g powders were dissolved in 20 ml of deionized water with magnetic stirring for 20 min to form a transparent solution. Then, the solution was transferred to a 50-ml Teflon-lined stainless-steel autoclave, which was heat-treated to 200ºC or 250ºC and held for 5 h. Next, the autoclave was cooled to room temperature naturally. The product was collected by centrifugation, and washed with deionized water three times and then dried at 90ºC for 6 h. The different molar ratios of ZrOCl_2_·8H_2_O and CH_3_COOK, i.e., 1:1.0, 1:1.5, and 1:2.0, were abbreviated Z1.0, Z1.5, and Z2.0, respectively.

### Material Characterization

X-ray diffraction (XRD) patterns for the samples heat-treated at different temperatures were recorded on a Bruker AXSD8 Advance X-ray diffractometer with Cu *K*α radiation using a graphite monochromator. Intensities of the diffraction peaks were recorded in the 10°-80° (2θ) range with a step size of 0.02°. The Fourier-transform infrared (FTIR) spectrum of the nanocrystals was recorded on a Nicolet 200sx FTIR spectrometer in the 4,000–400 cm^−1^ region using the KBr pellet method. The Raman spectrum was obtained using an NXR FT-Raman spectrometer with InGaAs as a detector at room temperature. The morphologies and microstructures of the nanocrystals were observed by scanning electron microscopy (SEM) using a Hitachi S-4800 instrument and high-resolution transmission electron microscopy (HRTEM) using a JEM-200CX instrument. The sample was placed in conductive adhesives and sprayed gold at 40 s in the process of SEM sample preparation and the powders were ultrasonic dispersed on the copper network for preparing HRTEM samples. The surface elemental composition and valent state of the nanocrystals were investigated by X-ray photoelectron spectroscopy (XPS) using a VG Scientific spectrophotometer with an X-ray source of Al *K*α radiation at 1,486.6 eV. The base pressure was approximately 10^–8^–10^–10^ Pa. The calibration of the *E*_*b*_ scale and the corrections for the *E*_*b*_ shift due to a steady-state charging effect were made by assuming that the C 1*s* line lies at 284.6 eV. The ZrO_2_ was ground into powder and then compressed into a pellet. The absorption spectrum was measured ultraviolet (UV) spectroscopy using a UV solution-U-3501 spectrophotometer. The steady-state fluorescence spectrum was obtained on an Edinburgh FLS920 fluorescence spectrometer equipped with a 450-W Xe lamp. The Brunauer–Emmett–Teller (BET) surface areas of the fibers were measured by N_2_ adsorption at 77 K with a Quadrasorb SI instrument. Fibers weighing approximately 40 mg were used for the measurements. Before the BET measurements, the samples were evacuated at 200ºC for 8 h in vacuum. The pore-size distribution was calculated by the density-functional-theory (DFT) method. The samples were pressed into a self-supporting thin wafer and put into the sample holder. The wafer was degassed in dynamic vacuum (10^−2^ Pa) at 573 K for 3 h, and then the background spectrum was recorded. After the equilibrium of adsorption pyridine vapor for 1 h, the spectrum was recorded after degassing the wafer in vacuum at 393 K for 2 h.

## Results and discussion

### Morphology, crystallinity and formation mechanism

**Figure 2:**
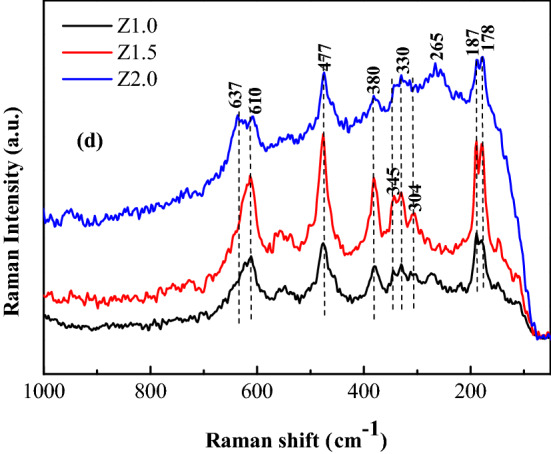
Raman spectra of ZrO_2_ nanocrystals heat-treated at 200ºC for 5.

Raman spectroscopy is a nondestructive experimental technique for probing the vibrational and structural properties of materials. It is also recognized as a powerful tool for identifying different polymorphs of metal oxides^28^. According to group theory, the monoclinic (*m*-ZrO_2_), tetragonal (*t*-ZrO_2_), and cubic (*c*-ZrO_2_) phases of ZrO_2_ are expected to have 18 (9*A*_*g*_ + 9*B*_*g*_), six (1*A*_1*g*_ + 2*B*_1*g*_ + 3*E*_*g*_) and one *T*_2*g*_ Raman-active modes, respectively. Figure 2 shows the Raman spectra as-obtained products at 200ºC, using a laser with wavelength 532 nm. According to Fig. 2, for Z2.0, peaks at 178 and 187 nm (*m*-ZrO_2_), 265 nm (*t*-ZrO_2_), 304 nm (*m*-ZrO_2_), 330 nm (*m*-ZrO_2_), 345 and 380 nm (*m*-ZrO_2_), 447 nm (*m*-ZrO_2_), 610 nm (*m*-ZrO_2_), and 637 (*t*-ZrO_2_) showed a mixture of monoclinic and tetragonal phases existing in the samples. For Z1.0 and Z1.5, only the peaks at 178 nm, 187 nm, 304 nm, 330 nm, 345 nm 380 nm, 477 nm, and 610 nm were observed. These peaks all come from monoclinic ZrO_2_, which indicates that there are no tetragonal phases ZrO_2_ in the samples.

**Figure 3:**
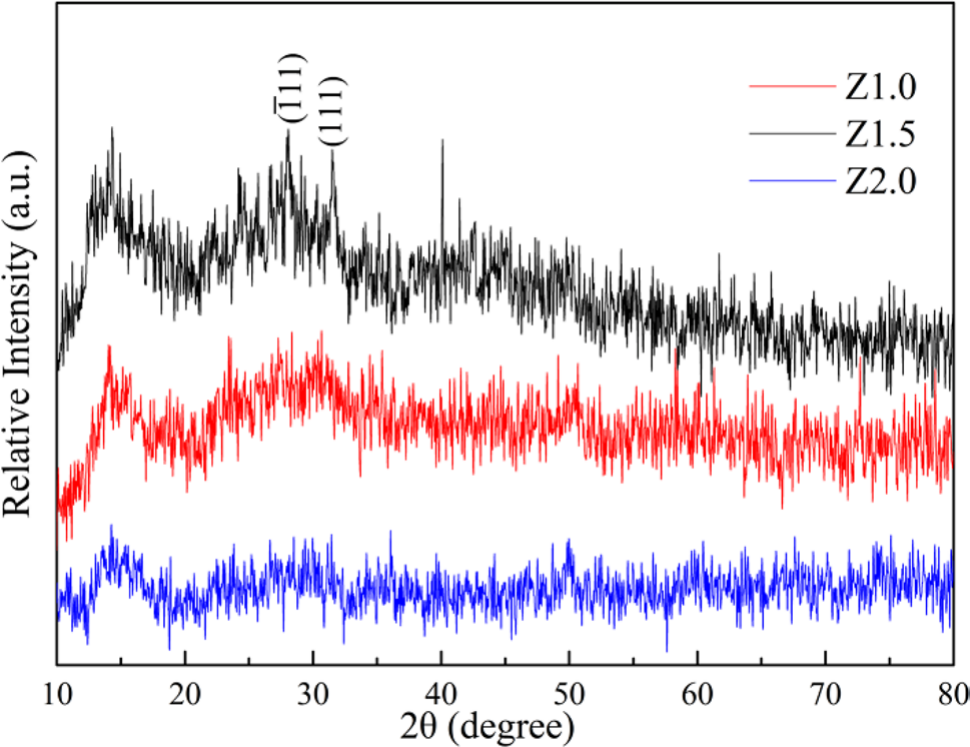
XRD patterns of ZrO_2_ nanostructures heat-treated at 200ºC for 5 h.

The XRD patterns of ZrO_2_ nanostructures heat-treated at 200ºC for 5 h are shown in Fig. 3. The diffraction peaks exhibited significant broadening, suggesting a small crystalline size of all the products. Among them, the Z1.5 sample had the best sharper peaks, and the ($\overline{1}$11) and (111) crystal faces of monoclinic zirconia (JCPDS 65-1025) are easily identified. Scherrer-line-width analyses of the (11) and (111) reflections gave an average crystalline size of 5.3 ± 0.2 and 10.0 ± 0.5 nm, respectively, indicating the small crystalline size and anisotropic morphology of the products, which were similar to the results of SEM and TEM, and all of which could be seen from the following results.

**Figure 4:**
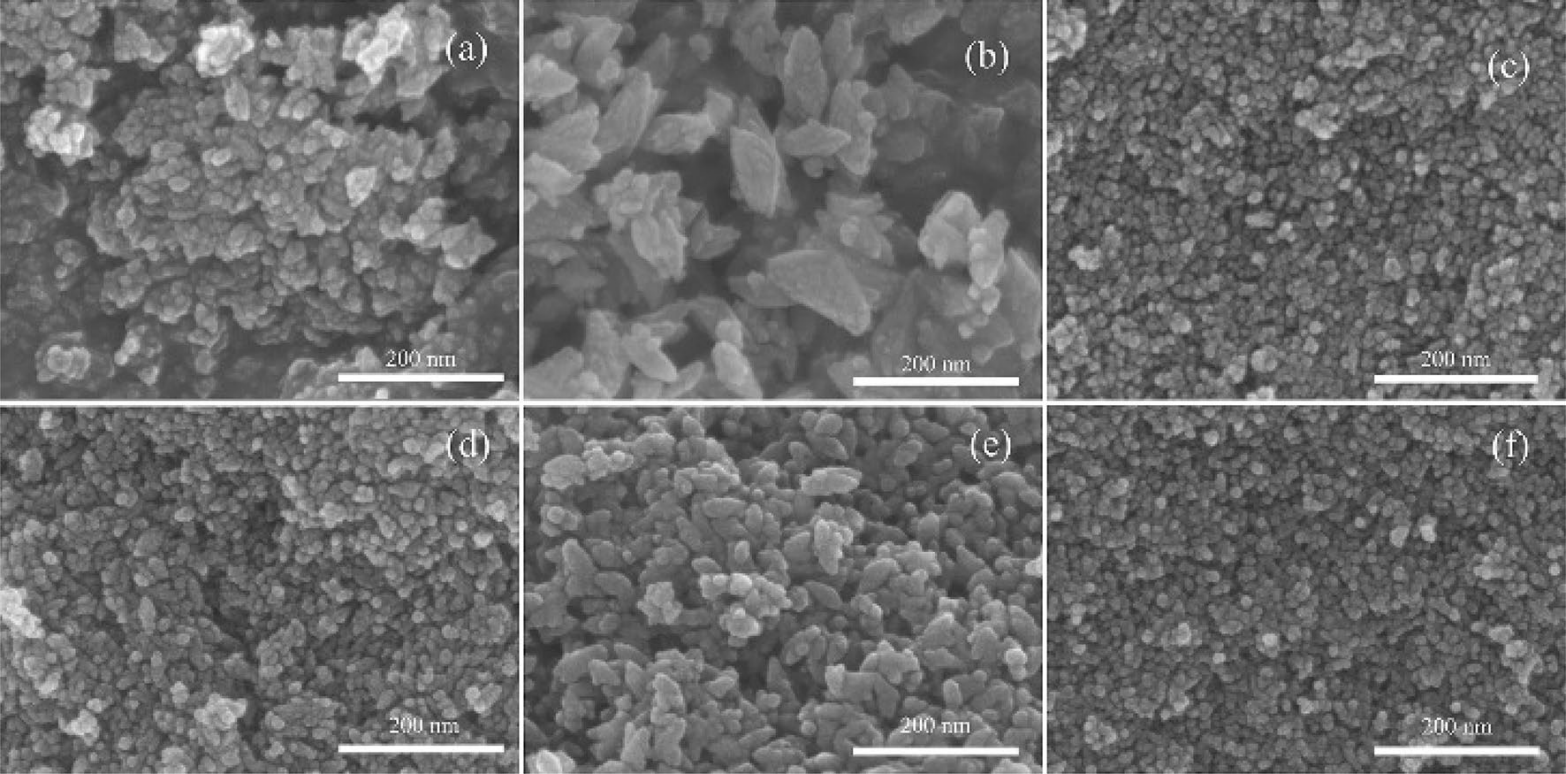
SEM images of ZrO_2_ nanostructures: (**a)** Z1.0, 200ºC; (**b)** Z1.5, 200ºC; (**c)** Z2.0, 200ºC; (**d)** Z1.0, 250ºC; (**e)** Z1.5, 250ºC; (**f)** Z2.0-250ºC.

The morphology and microstructure were characterized by SEM and HRTEM. The SEM images (Fig. 4) showed that the nanoparticles exhibited different structures. The ZrO_2_ spheres were observed for Z1.0 and Z2.0, but for that of Z1.5, the anisotropic star-like ZrO_2_ structures with a wide size distribution from 40 to 100 nm. Another interesting phenomenon was that the average crystal size $\overline{1}$ was decreased with the temperature increasing from 200ºC to 250ºC.

**Figure 5:**
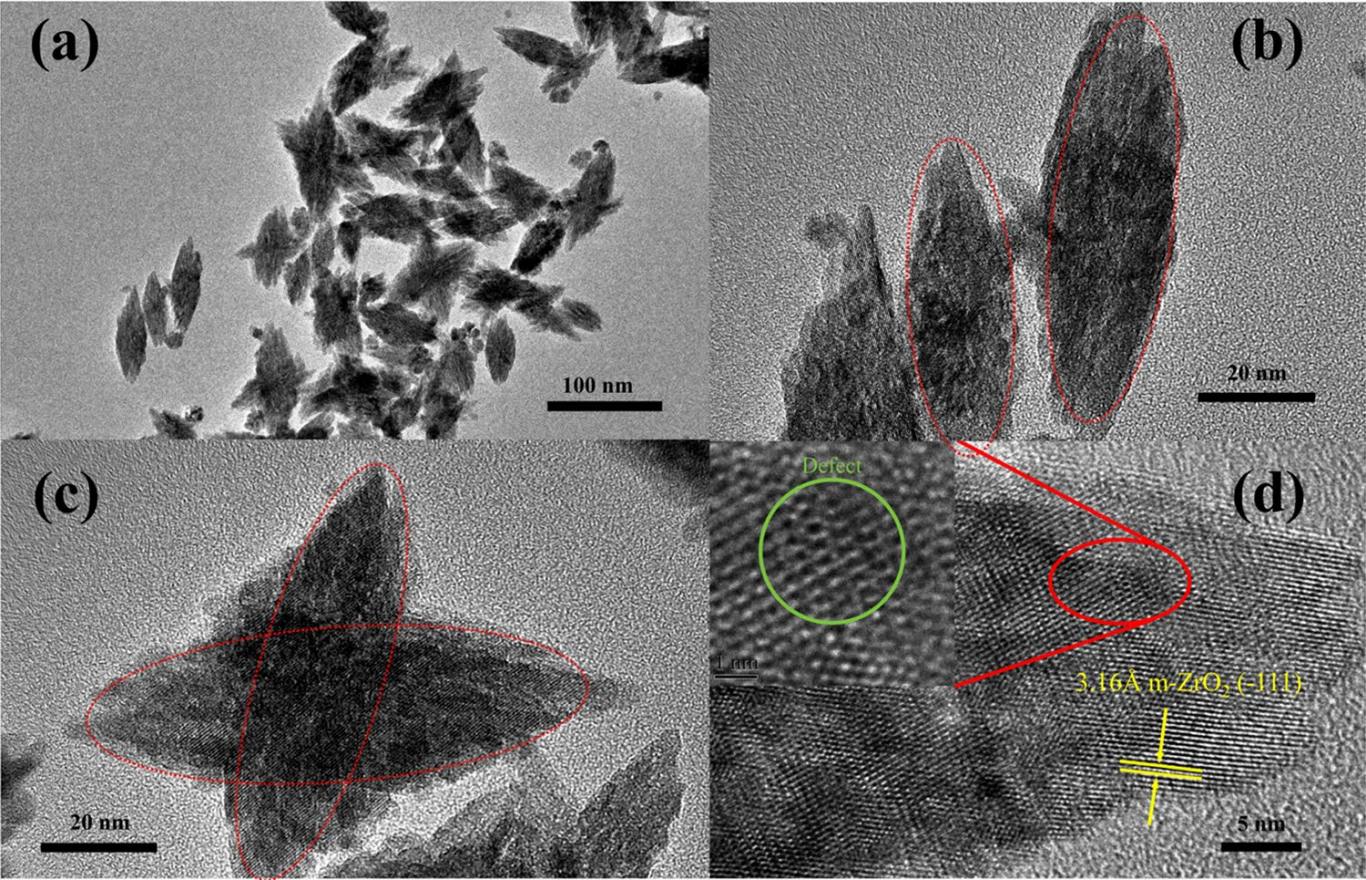
HRTEM images of (**a)** elongated platelets and (**c)** star-like nanostructures of Z 1.5–200ºC; (**b**,**d**) are high magnification views of (**a**,**c**), respectively.

**Figure 6:**
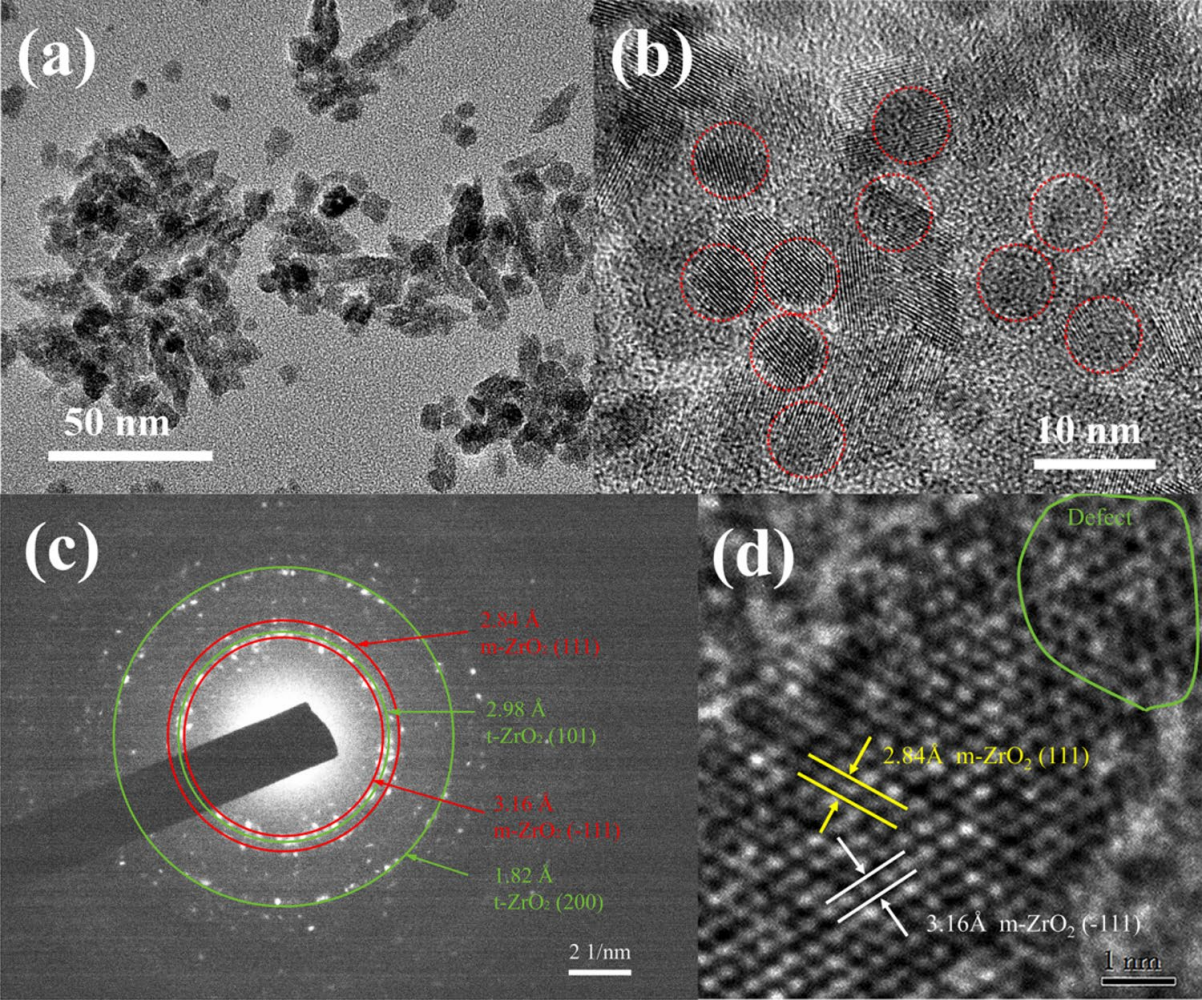
HRTEM images at different magnifications of (**a**) and (**b**) spherical nanoparticles, (**c**) electron powder-diffraction pattern of Z 2.0–200ºC, and (**d**) HRTEM image of spherical nanostructures.

The HRTEM images (Figs. 5 and 6) revealed that the nanostructures possessing high crystallinity. As can be seen from Figs. 4c, 5b, the uniform elongated platelets and star-like structures were composed of short nanorods with a diameter of approximately 5 nm and length of approximately 10 nm. This confirmed that the nanocrystals composed of star-like ZrO_2_ nanostructures were anisotropic. For the star-like nanostructures, the two particles were super-positioned with a special angle like the butterfly, which were different from the previous report^8^. Furthermore, some nanopores existed at the interface, which should be caused by the particles’ aggregation. From Fig. 5d, the lattices were clear, and the measured results showed that the lattice spacing 3.16 Å, which matched perfectly with the ($\overline{1}$ 11) of m-ZrO_2_. In addition, there are some lattice defects between the lattices.

Figure 6a,b showed the spherical agglomerated ZrO_2_ particles in the size range of 5–6 nm. The corresponding electron powder-diffraction pattern (Fig. 6c) was presented at least four diffraction rings, which match the ($\overline{1}$11), (111) lattice plane of m-ZrO_2_ and (101), (200) lattice plane of t-ZrO_2_ (JCPDS 42-1164), respectively. This result is consistent with the Raman spectra. In the Fig. 6d, multiple sets of lattice fringes can be seen. Each set of lattice fringes is parallel to each other, and the interplanar spacing is 3.16 Å and 2.84 Å, respectively, corresponding to the ($\overline{1}$11) and (111) plane of m-ZrO_2_. Same as the sample Z1.5, the sample Z2.0 also have some lattice defects.

**Figure 7:**
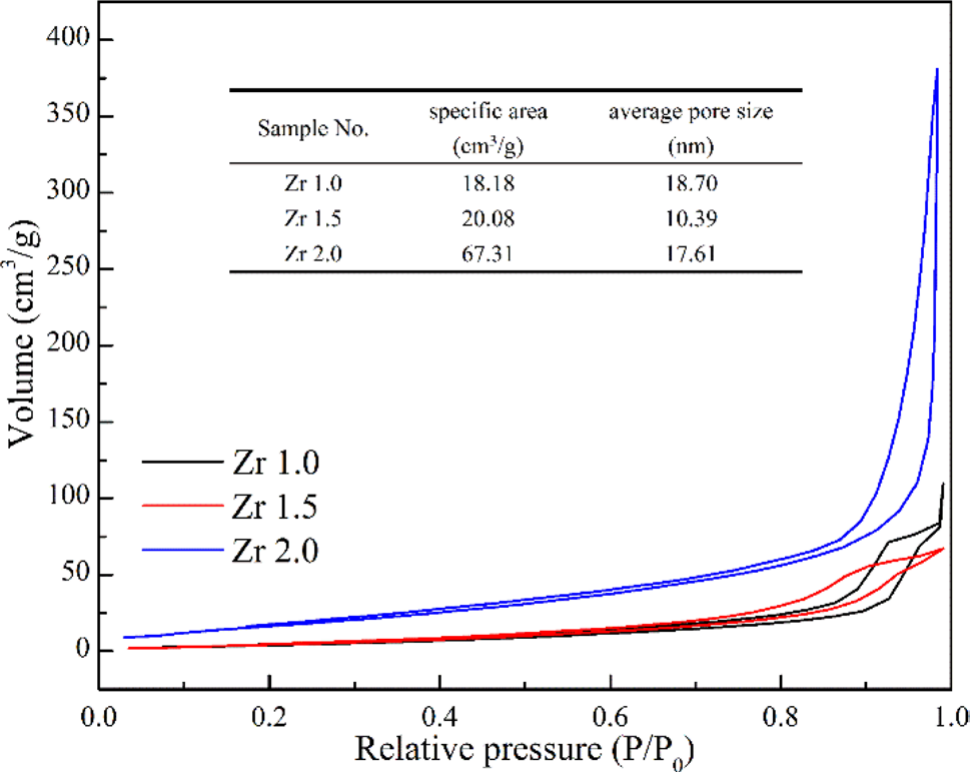
N_2_ adsorption and desorption isotherms of ZrO_2_ nanoparticles.

The N_2_ adsorption and desorption isotherms of the ZrO_2_ nanoparticles are shown in Fig. 7. Through calculation of the BET equation, the resultant BET specific surface areas were 18.18, 20.08, and 67.31 m^2^/g for Z1.0, Z1.5, and Z2.0, respectively. The specific surface of Z2.0 is larger than Z1.0, which was caused by the particle size of Z2.0 is smaller than Z1.0. The result was consistent with the previous analysis.

**Figure 8:**
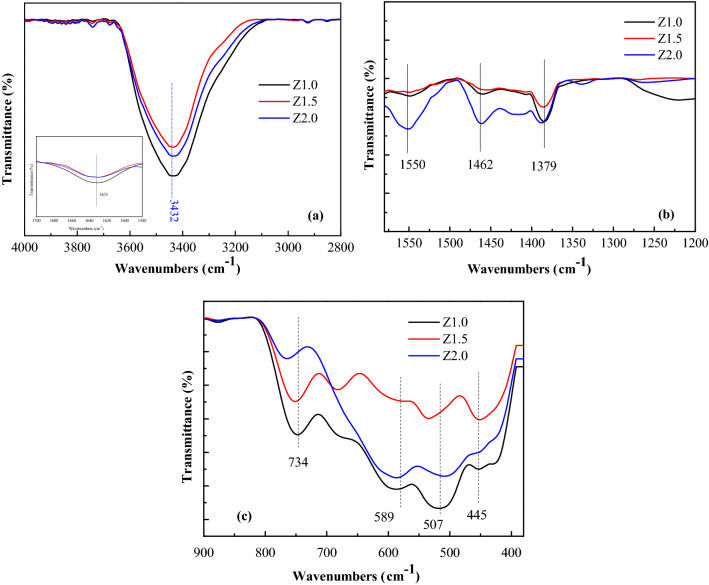
IR spectra of ZrO_2_ nanocrystals heat-treated at 200ºC for 5 h.

In Fig. 8, the absorptions at 3,432 and 1633 cm^−1^ were ascribed to the -OH vibrations of the adsorbed water or the surface hydroxyls. For the samples, the vibration at 1,550 cm^−1^ was attributed to the symmetric vibration absorption of COO^−^, and those at 1,462 and 1,379 cm^−1^ were due to the asymmetric vibration absorptions of COO^−^, indicating the existence of acetate on the surface of the product. The position and separation (Δ) of COO^−^ bands in the 1,300–1,700-cm^−1^ region could be used to deduce the carboxylate coordination mode^29^. In this case, the Δ values [ν_*a*_(COO^−^)-ν_*s*_(COO^-^)] are 88 cm^−1^ (1,550–1,462 cm^−1^) and 171 cm^−1^ (1,550–1,379 cm^−1^), revealing unidentate coordination and bridging coordination modes between COO^−^ and the surface Zr^4+^. The absorptions at 734, 589, 507, and 455 cm^−1^ were the vibrations of Zr-O. It could be concluded from the IR spectrum that absorbed water, hydroxyl, and acetate groups were on the surface of the products. The difference was that the absorption peaks in the region 1,300–1,600 cm^−1^ of the sample Z2.0 are very weak or are barely even visible.

**Figure 9:**
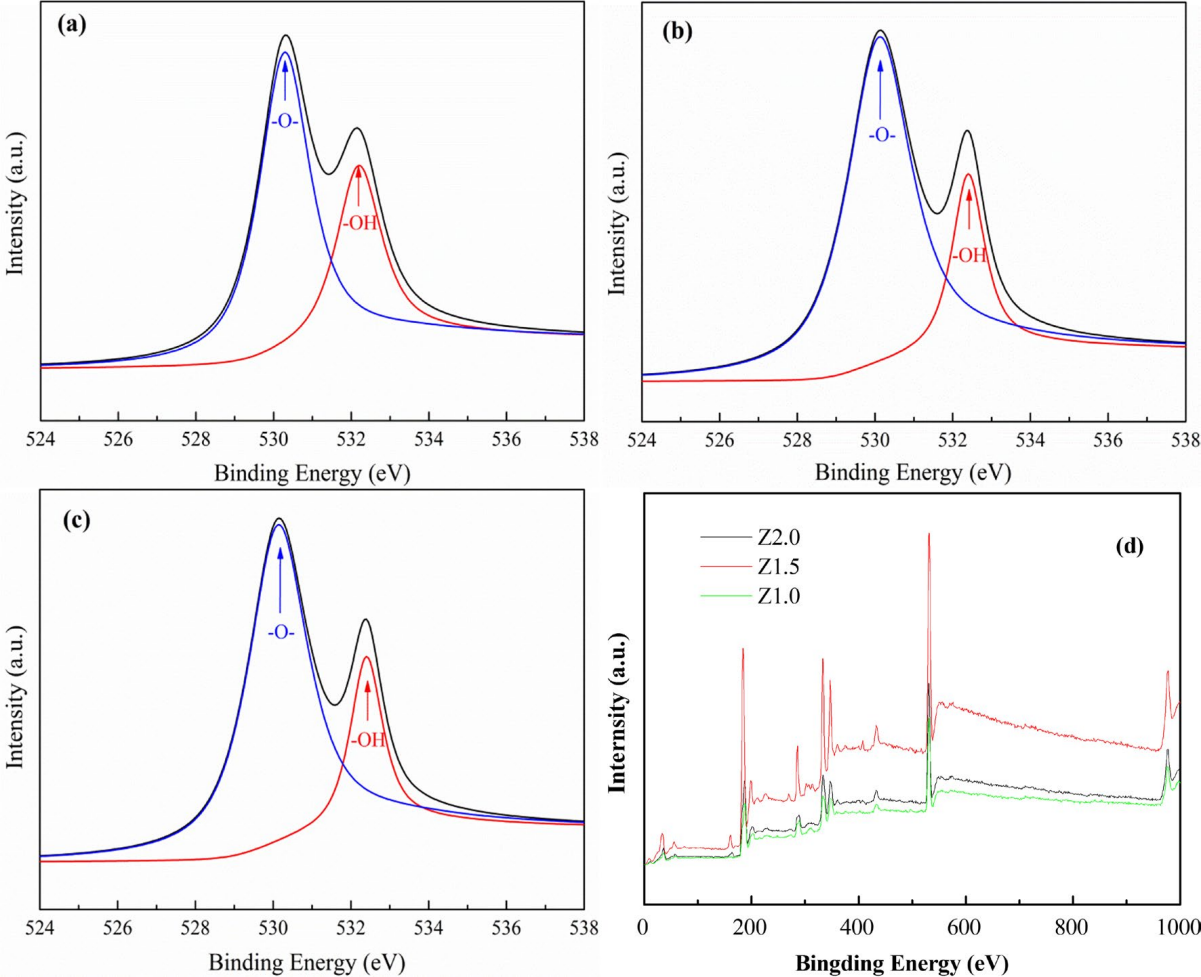
–O– and –OH on the surface of ZrO_2_ nanocrystals heat-treated at 200ºC for 5 h.

The XPS measurements technique is often used for observing the variation in surfaces applied for catalysis, including the oxidation of Zr. The XPS spectra of O 1*s* in Fig. 9 are wide and asymmetric, with the left side wider than the right, which can be seen from the figure, indicating that at least two kinds of O species were present at the surface, which could be recognized by resolving the XPS curves. The dominant peak at approximately 529.6 eV is a characteristic of lattice O in samples, while the signal at 532.6 eV can be associated with surface hydroxyl groups^30^. Based on the area integral of the two peaks corresponding to lattice O (OL) and surface hydroxyl (-OH) of O 1*s* photoemissions, the ratio of -OH to OT (OT denotes total oxygen, OT = –OH + OL) for all samples was calculated as shown in Table 1. It is worth noting that the content of CH_3_COO^−^ species could effectively enhance the surface hydroxyl group on the surface of the ZrO_2_ nanocrystals. With the increasing addition of CH_3_COO^−^ species, the amount of surface hydroxyl groups increased.

**Table 1:** Binding-energy values of lattice oxygen (OL) and surface hydroxyl (-OH) and the ratio of -OH to OT for samples heat-treated at 200ºC for 5 h.

Sample	Binding energy (eV)		Ratio of –OH to OT (%)
	OL	–OH	
Z1.0	529.6	532.6	12.3
Z1.5	529.6	532.8	14.2
Z2.0	532.6	532.6	18.0

**Figure 10:**
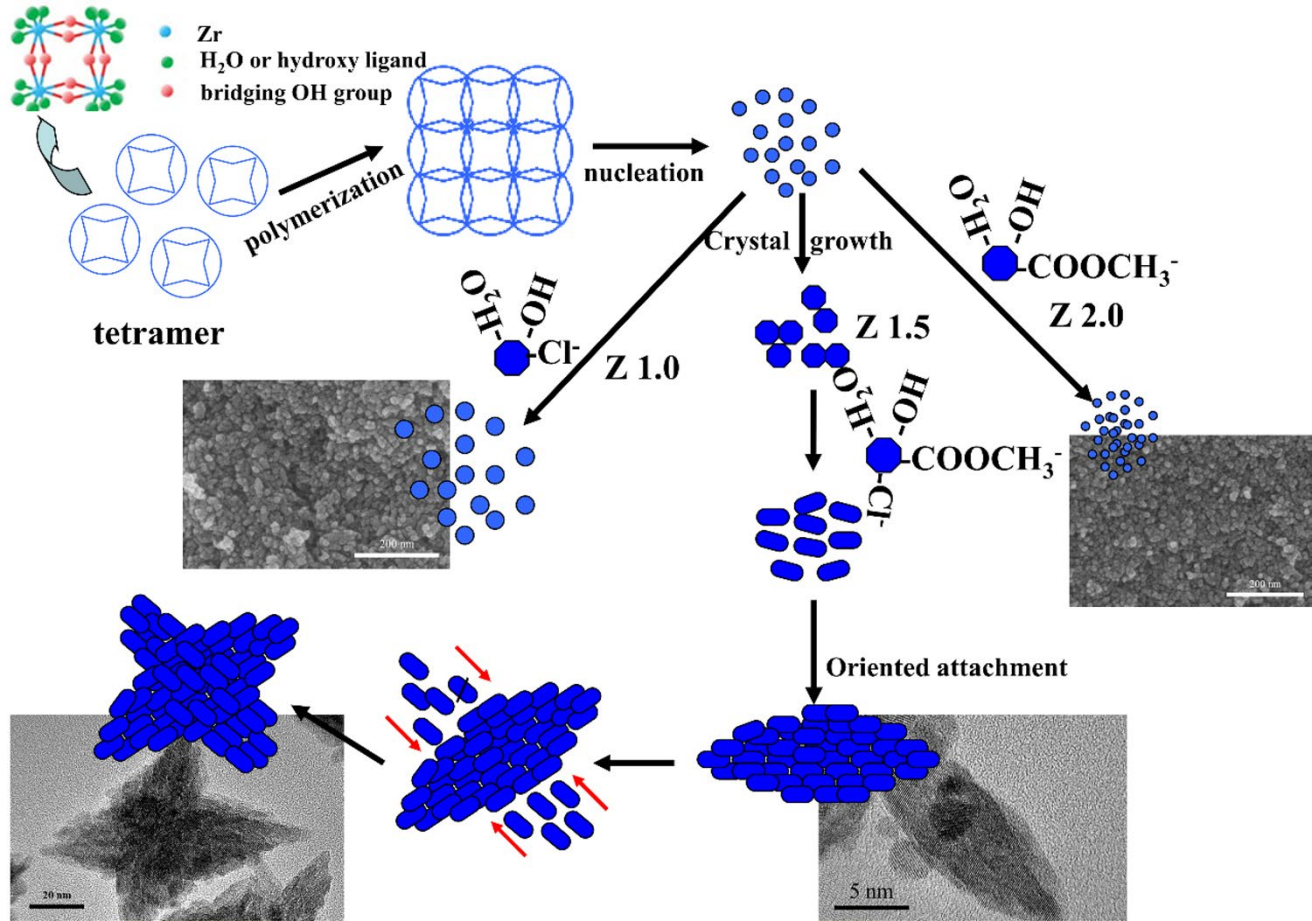
Schematic illustrating the formation of ZrO_2_ nanoparticles with different morphologies.

The schematic formation of ZrO_2_ nanoparticles with different morphologies is illustrated in Fig. 10. ZrOCl_2_∙8H_2_O forms a tetramer complex in methanol solution with a structure in which four Zr atoms are arranged in a square, and each Zr atom is coordinated by four bridging -OH groups and four H_2_O molecules or hydroxyl ligands, and then goes through a polymerization process by a dehydration reaction to form polymeric species^31^. When the concentration of polymeric species reaches the critical supersaturation level, ZrO_2_ crystal nuclei form spontaneously in the aqueous solution, and then evolve into primary ZrO_2_ crystals. The grain growth is affected by the conditions, such as pH value, anions, etc., very obviously. The different reaction equations between ZrOCl_2_∙8H_2_O and CH_3_COOK with different molar ratios are expressed as follows:

$$\begin{aligned} & {\text{ZrOCl}}_{{2}} \cdot {\text{8H}}_{{2}} {\text{O }} + {\text{ CH}}_{{3}} {\text{COOK }} \to {\text{ Zr}}\left( {{\text{CH}}_{{3}} {\text{COO}}} \right)\left( {{\text{OH}}} \right)_{{2}} {\text{Cl }} + {\text{ KCl}} \downarrow \, + {\text{ 7H}}_{{2}} {\text{O}}, \\ & {\text{ZrOCl}}_{{2}} \cdot {\text{8H}}_{{2}} {\text{O }} + {\text{ 2CH}}_{{3}} {\text{COOK }} \to {\text{ Zr}}\left( {{\text{CH}}_{{3}} {\text{COO}}} \right)_{{2}} \left( {{\text{OH}}} \right)_{{2}} + {\text{ 2KCl}} \downarrow \, + {\text{ 7H}}_{{2}} {\text{O}}. \\ \end{aligned}$$

According to the IR results, each Zr atom was coordinated by one CH_3_COO^−^ in the structure of the precursor. For Z1.0, besides CH_3_COO^−^, the same molar amount of Cl^−^ existed in the aqueous solutions, and only CH_3_COO^−^ was contained in the Z2.0 system. The anions can be considered as “modifiers” that control the morphology and crystal sizes of the ZrO_2_ nanoparticles. In contrast, the volume and viscosity of the CH_3_COO^−^ were larger than those of the Cl^−^, which may have more resistance for grain growth. Therefore, the smaller grain size was observed for the Z2.0 system compared with that of Z1.0. When the two anions existed in one system simultaneously for Z1.5, the primary ZrO_2_ nanoparticles coalesced with each other to form the rod-like ZrO_2_ through oriented attachment (OA) due to the synergy of the anions, which has been already proved to be an important mechanism for the anisotropic growth of nanostructures.

### Optical properties

**Figure 11:**
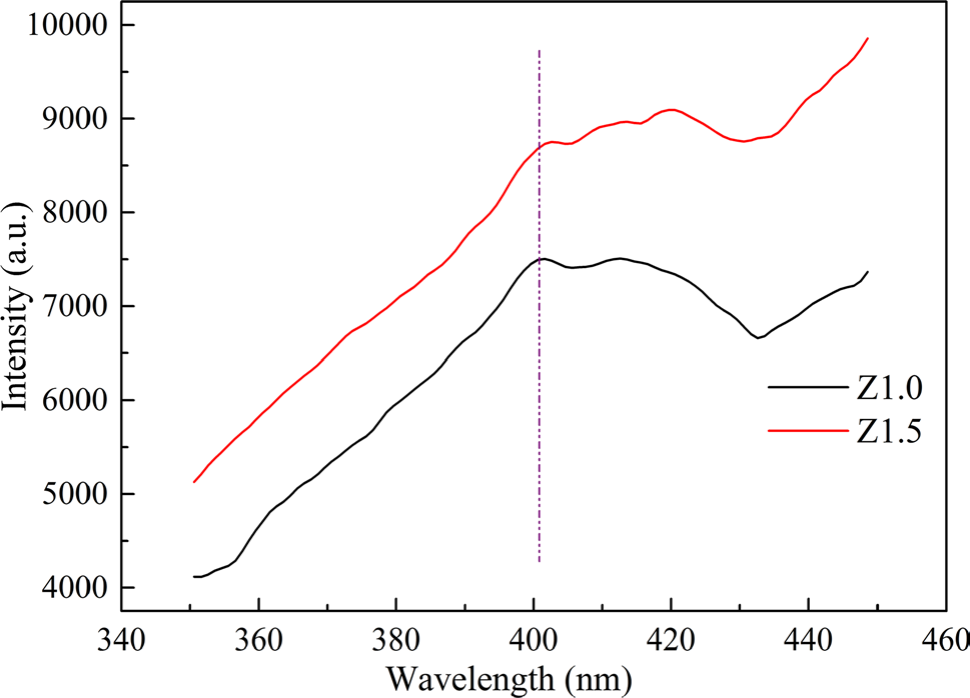
Fluorescence emission spectra excited at 260 nm for ZrO_2_ nanoparticles for Z1.0 and Z1.5 heat-treated at 200ºC for 5 h.

Fluorescence spectra were measured with several wavelengths between 245 and 290 nm, while the fluorescence intensity changed to some extent with excitation wavelength. The fluorescence band position and the band shape remained approximately the same for excitation wavelengths below 290 nm and the different morphologies of the nanoparticles. Figure 11 shows the representative fluorescence emission spectra excited at 260 nm for Z1.0 and Z1.5 heat-treated at 200ºC for 5 h, which featured a broad fluorescence band centered at 400 nm. This broad band (~ 3.1 eV) and the substantial redshift of the band maximum compared to the band gap (~ 5.6 eV) of the bulk material^32^ strongly indicated that the fluorescence involved extrinsic states. Because the particle-size distribution was very narrow, the broad fluorescence band seemed to be mostly caused by the small particle size, which led to an inhomogeneous broadening from a distribution of the surface or defect states. The fluorescence intensity of Z1.5 is stronger than Z1.0, which could be ascribed to the increasing of the crystallinity as shown in the XRD analysis in Fig. 3.

## Conclusions

ZrO_2_ nanocrystals with different morphologies were prepared by the hydrothermal method. The morphology, crystallinity, and optical property of as-synthesized nanoparticles were characterized using SEM, HRTEM, XRD, Raman spectroscopy, PL spectroscopy, and BET measurements. Both CH_3_COO^−^ and Cl^−^ greatly affected the crystal size, phases, and morphologies of the ZrO_2_ nanoparticles. Under the reaction conditions used in this work, the morphology and crystallinity of the resulting ZrO_2_ nanoparticles could be adjusted. The nanoparticles exhibited photoluminescence in the UV region, which suggested that the as-synthesized ZrO_2_ nanoparticles may find use as luminescent labels and light-emitting molecular substances in nanoscale material fields.
